# Intravenous Immunoglobulin and Mycophenolate Mofetil for Long-Standing Sensory Neuronopathy in Sjögren's Syndrome

**DOI:** 10.1155/2012/186320

**Published:** 2012-11-22

**Authors:** Maria Giovanna Danieli, Lucia Pettinari, Ramona Morariu, Fernando Monteforte, Francesco Logullo

**Affiliations:** ^1^Clinica Medica, Dipartimento di Scienze Cliniche e Molecolari, Università Politecnica delle Marche & Ospedali Riuniti, Via Tronto 10, 60126 Ancona, Italy; ^2^U.O. di Medicina-LPA, Presidio di Loreto, 66025 Loreto, Italy; ^3^U.O. di Radiodiagnostica, Ospedale di Casarano, 73042 Lecce, Italy; ^4^Clinica Neurologica, Dipartimento di Medicina Sperimentale e Clinica, Polo Didattico Scientifico, Università Politecnica delle Marche & Azienda Ospedali Riuniti, Via Tronto 10, 60126 Ancona, Italy

## Abstract

Sensory neuronopathy is described in association with the Sjögren's syndrome (SS). We studied a 55-year-old woman with a 4-year history of progressive asymmetric numbness, distal tingling, and burning sensation in upper and lower limbs. In a few months, she developed ataxia with increased hypoanaesthesia. Electrodiagnostic tests revealed undetectable distal and proximal sensory nerve action potential in upper and lower limbs. Cervical spine magnetic resonance showed a signal hyperintensity of posterior columns. Previous treatment with high-dose glucocorticoids and azathioprine was ineffective. A combined treatment with intravenous immunoglobulin and mycophenolate mofetil was followed by a progressive and persistent improvement. This case documented the efficacy and the safety of the coadministration of intravenous immunoglobulin and mycophenolate mofetil in sensory neuronopathy associated with SS refractory to conventional immunosuppressive therapy.

## 1. Introduction

Central nervous system involvement in Sjögren's syndrome (SS) is rarely reported and may be severe and varied [1]. Sensory neuronopathy (or sensory ganglionopathy, SN) is a distinctive neuropathy of SS, accounting for 15–20% of all neuropathies seen in this condition [2]. A sensory neuropathy is often the presenting feature of SS, and, therefore, a high index of suspicion is required, particularly in female patients with non-length-dependent, painful, or ataxic sensory neuropathy or those with trigeminal sensory and autonomic involvement [3]. At the onset of SN, numbness, tingling, burning, and pain sensations are reported in all limbs, usually with asymmetric distribution. With the disease progression, the sensory disturbances can involve the trunk, the face or they develop into a symmetric way. On examination, degeneration of large sensory neurons leads to gait ataxia, proprioceptive sensory loss, and widespread deep tendon areflexia [3]. When smaller sensory neurons are affected, deficits are those of hypoesthesia to pain and thermal stimuli with hyperacute pain. Autonomic nervous system involvement may cause fixed tachycardia, orthostatic hypotension, and gastrointestinal pseudo-obstruction. The response to treatment is usually poor, even with glucocorticoids, immunosuppressants, and plasmapheresis [3].

Here we report the case of a woman with primary SS who presented with SN that was successfully managed with intravenous immunoglobulin and mycophenolate mofetil coadministration.

## 2. Case Report

In 2001, a 55-year-old woman presented with progressive asymmetric numbness distal tingling and burning sensation in upper limbs associated with xerostomia and xerophtalmia. Antibodies to SS-A/Ro and anti-SS-B/La were positive. A minor salivary gland biopsy showed mononuclear cells with prominent lymphocyte infiltration with glandular cell atrophy. Nerve conduction studies showed a “sensory axonal neuropathy.” The diagnosis of SS was made according to the criteria of American-European Community [4], and she was treated with anti-inflammatory drugs. In 2003, distal sensory deficits aggravated and extended to the lower limbs with increased hypo-anaesthesia and unsteady gait. In spite of treatment with oral prednisone (1 mg/kg/day) and azathioprine (2 mg/kg/day), distal sensory deficits progressed. Thus, she was admitted to our hospital in June, 2005.

On admission she was bedridden and she could not ambulate independently. A global impairment of sensation was detected as a profound loss in all lower limbs and, as moderate reduction, in the upper limbs. Deep tendon reflexes were absent. No autonomic symptoms were detected. Neurological examination of the cranial nerves was normal. Muscle strength was normal in all of the four limbs. Severe sensory ataxia was present in assisted gait. Romberg's sign was positive.

**Figure 1:**
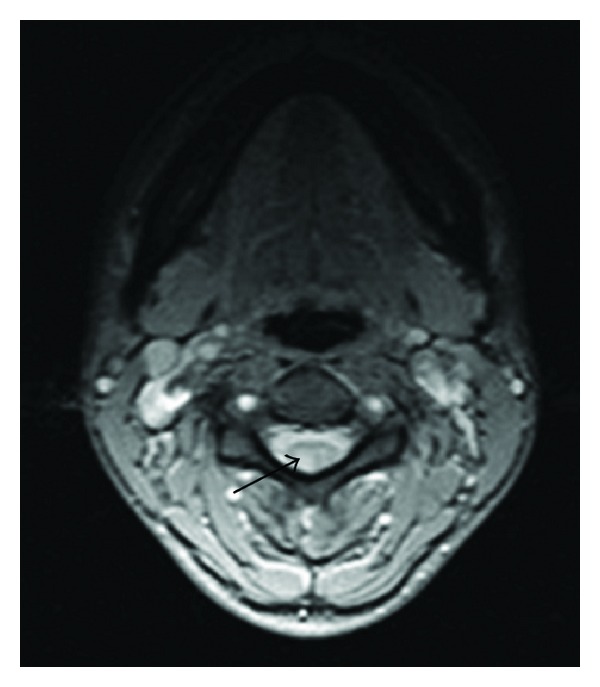
MRI 1.5T Axial section obtained with sequence GRE-T2 at C4 level showing a signal hyperintensity of posterior columns.

We documented a mild normocytic anaemia with lymphopenia with high erythrocyte sedimentation rate. The antinuclear antibody titre was elevated with positive anti-SS-A/Ro and anti-SS-B/La by fluorescence enzyme immunoassay. Levels of immunoglobulins (IgG, IgM, and IgA) and serum concentrations of complement levels (C3 and C4) measured by nephelometry were normal. As for serological autoimmune markers, immunofixation did not detect monoclonal immunoglobulins; cryoglobulins were negative, as ANA and rheumatoid factors (IgM-RF) and anti-CCP antibodies. HBV and HCV markers were negative. Electrodiagnostic studies revealed undetectable distal and proximal sensory nerve action potential (SNPAs) in upper and lower limbs. Nerve conduction studies were normal. Concentric needle examination of distal and proximal muscles was normal. Somatosensory-evoked potentials were absent with distal stimulation, both in upper and lower limbs. Spinal cord magnetic resonance disclosed high signal intensity without gadolinium enhancement in posterior columns of the cervical spinal cord (Figure 1), findings consisting with the diagnosis of neuronopathy.

We started a combined treatment with intravenous immunoglobulin and oral mycophenolate mofetil. Intravenous immunoglobulin was infused at 1 g/kg/day (5 g/hour) on two consecutive days each month for six months, followed by further cycles every other month for six months. Oral mycophenolate mofetil was started at 500 mg/day and then titrated to the definite dosage of 30 mg/kg/day. Oral prednisone was slowly tapered from the initial dose of 1 mg/kg/day to an average of 0.25 mg/kg every other day.

Within three months, the patient presented a marked improvement in sensory symptoms, in gait and in the functional status. No modification of *sicca syndrome* was reported. She continued with MMF for one year more and with IVIg with decreased doses and longer intervals between courses for two years. At last followup she was ataxic but she can ambulate without support, and the sensory loss in upper and lower limbs was notably reduced. Magnetic resonance features were unchanged.

## 3. Discussion

We report a case of a long-standing severe ganglionopathy in the context of SS for which the combined treatment with intravenous immunoglobulin and mycophenolate mofetil was remarkably and persistently successful. The pathogenic mechanism responsible for the neuronopathy is still debated. Some authors have postulated that the dorsal root ganglion damage is associated with loss of neurons and mononuclear inflammatory infiltrates without vasculitis [5]. In the literature, few studies, mainly case reports or small series, investigated the therapeutic options for neuronopathy. More studies are available for nonvasculitic neuropathies associated with SS. For both forms, the response to traditional glucocorticoids and immunosuppressants is generally poor [5]. Cyclophosphamide, used preferentially in vasculitic neuropathies, is linked to a certain degree of toxicity. In sensory neuropathy associated with SS, positive results have been reported following the use of IVIg, with long-term sustained improvement and a reduction in the rate and severity of glucocorticoids-related adverse effects [6, 7]. In a retrospective national multicentric study, Rist et al. [8] documented the benefit of IVIg in 19 patients with SS-related neuropathy without any necrotizing vasculitis. The clinical response was observed after two courses of IVIg administration, thus underlying the necessity to evaluate the treatment response only after two courses.

As for MMF in SS, we found in the literature only a small pilot trial in patients refractory to other immunosuppressants. The authors documented an improvement in xerophthalmia as well in some laboratory parameters (reduction of gamma globulins and IgM-RF titre, increase of C3 and C4 complement levels and white blood cell count) [9].

No data are available on the use of coadministration of intravenous immunoglobulin and mycophenolate mofetil in SS. In neuronopathy, the rationale of using intravenous immunoglobulin and mycophenolate mofetil involves the cell-mediated [5] and humoral [10] immunological mechanisms underlying the ganglionopathy. Immunoglobulin is widely used in autoimmune diseases, intravenously and subcutaneously [11, 12]. Among other mechanisms, intravenous immunoglobulin can affect T regulatory cells by increasing their suppressive function [13] and accelerate the rate of the pathogenic IgG catabolism [11, 14]. Mycophenolate mofetil affects the *de novo* synthesis of guanosine nucleotides thus inhibiting a crucial pathway for DNA synthesis in lymphocytes. The synergism between the action of intravenous immunoglobulin and mycophenolate mofetil in suppressing the activation and the proliferation of lymphocytes could explain the favourable response and the long-term remission we observed.

We choose to continue the IVIg administration at monthly intervals, since in previous reports only repeated doses of IVIg permitted the maintenance of the clinical response [6]. Moreover, the use of IVIg enables a reduction of the infective risk in subjects treated with immunosuppressant. This point is of particular interest since immunodeficiency states are increasingly recognised in patients with immune-mediated diseases and they are due to intrinsic defect linked to the disease itself and/or to the immunosuppressant employed throughout the disease management.

In conclusion, we documented a distinct improvement of the disability linked to ganglionopathy within the first three months of intravenous immunoglobulin coadministered with mycophenolate mofetil treatment. The better neurological condition was maintained over a period of more than 4 years. The coadministration of intravenous immunoglobulin and mycophenolate mofetil was demonstrated to be beneficial and safe in a case of sensory neuronopathy associated with SS refractory to conventional immunosuppressive therapy.

**Take Home Messages**

- Intravenous immunoglobulin has been widely used in the treatment of immune-mediated diseases.
- Not many reports described the intravenous immunoglobulin application in Sjögren's syndrome, and even fewer are related to mycophenolate mofetil.
- The use of intravenous immunoglobulin co-administered with mycophenolate mofetil permitted to attain a complete remission with a relevant functional recovery in a subject with long-standing refractory neuronopathy.
- No side effects were linked to this combined treatment.
